# Exploring the Relation Between Diabetes and HIV: A Narrative Review

**DOI:** 10.7759/cureus.43909

**Published:** 2023-08-22

**Authors:** Mayank Kumar, Harshit Singh, Swarupa Chakole

**Affiliations:** 1 Community Medicine, Jawaharlal Nehru Medical College, Datta Meghe Institute of Higher Education and Research, Wardha, IND

**Keywords:** nuclease inhibitors, lipo dystrophy, antiretroviral therapy, hiv, diabetes mellitus

## Abstract

Diabetes mellitus, more usually abbreviated as DM or just diabetes, is a devastating metabolic disorder that claims many lives every year. Due to various variables, including the aging of the HIV (human immunodeficiency virus)-infected population and the high prevalence of chronic medical conditions among persons living with HIV, the crossroads of DM and HIV infection has become a significant research topic. Although the connection between HIV and diabetes is not simple, many aspects of the virus and its treatment have been connected to the onset of diabetes. The presence of inconclusive evidence that HIV is a risk factor for diabetes makes this area more challenging and debatable. This article examines the prevalence of DM in the HIV-positive community, along with its assessment, management, and treatment objectives. The most recent diabetes treatment recommendations from authoritative groups are considered in this article to give readers thorough and current advice. These guidelines emphasize the importance of tailoring pharmacological therapy and treatment goals to suit the specific needs of individuals with diabetes, including those who are also living with HIV. Individualizing treatment plans ensures that healthcare professionals consider comorbidities, medication interactions, and potential side effects when managing diabetes concerning HIV. In the later part of the article, a holistic approach is discussed to address the increased risk of cardiovascular disease and associated complications in HIV-positive individuals with diabetes. This approach aims to mitigate cardiovascular risks and improve overall health outcomes through comprehensive strategies such as lifestyle modifications, regular monitoring, medication management, and integration of multidisciplinary healthcare teams. By considering the unique challenges and considerations of individuals living with both HIV and diabetes, healthcare providers can develop targeted interventions and provide optimal care. In order to improve the life and health of persons living with HIV and diabetes, the article stresses the significance of cooperation amongst professionals in these fields.

## Introduction and background

Diabetes mellitus (DM), characterized by hyperglycemia due to decreased insulin production and sensitivity [1], is a chronic condition. About 80% of those affected reside in poor and developing countries [2], out of a total of 43 crores people around the globe. This number is approximated to reach 65 crores by 2045. There are almost two-thirds as many persons with diabetes who have not been diagnosed as yet [2]. In addition to well-known risk factors, including lack of exercise and poor diet, novel risk factors, such as persistent infections/inflammation, may be responsible for the observed changes. It has been hypothesized that HIV and treatments for it may be responsible. Despite concerted efforts to curb its spread, the acquired immunodeficiency syndrome (AIDS) pandemic, which is about to enter its 40th year, profoundly affects people's lives, households, and national economies. According to estimates, 3.6 crore persons worldwide had HIV that same year. They include 3.4 crore adults [3]. The availability of effective treatments is another focus of efforts alongside prevention. As of June 2017, about 2.1 crore people thriving with HIV had undergone antiretroviral treatment (ART), a significant rise from 1.7 crore in 2015 and 80 lakhs in 2010 [3].

The number of persons in poor nations taking ART for HIV increased by 13 between 2004 and 2009. As a result, the fatalities have decreased, and those affected are surviving longer than ever. Longer life expectancy for people with HIV in developing countries is followed by a rise in non-communicable diseases (NCDs) such as cardiovascular disease and DM [4]. Substantial data have been collected, demonstrating that the incidence of non-communicable diseases among persons thriving with HIV will continue to grow, regardless of the natural evolution of the virus, related co-infections, or medications. Type 2 diabetes and metabolic derangement have been connected to HIV infection and ART [5]. HIV is also said to be cognate with an elevated risk of insulin resistance. Patients infected with HIV often have different infections, such as tuberculosis (TB) or viral hepatitis, which are also associated with a higher chance of the ailment [6-7].

With the current double burden of ailments, it is essential to completely understand how to handle both HIV infection and diabetes simultaneously. As well as discussing the research on the incidence and causes of this relationship, this article will explore the treatment implications of HIV's association with insulin resistance and diabetes.

## Review

Methodology

The search involved using a database, such as 'PubMed,' to explore terms like 'diabetes,' 'HIV,' 'protease inhibitors,' 'antiretroviral therapy,' 'nuclease inhibitors,' and 'lipodystrophy.' In this case, only English-language results were shown. When more than one report of a single study was discovered in the literature, the most current one was chosen. We only considered review papers that had some new findings.

Epidemiology of DM in HIV-infected patients

Studies have found 3%-15% of HIV-positive patients also have diabetic mellitus [8-11]. Possible explanations for this mismatch include differences in the study population, the method employed to diagnose DM, and the presence of confounding variables. While some research suggests that people thriving with infection are extra prone to develop type 2 diabetes [8, 12-13], other studies have revealed either no association between HIV and type 2 diabetes or even the opposite impact [10, 14-15].

Although there is conclusive evidence linking age, obesity, and a family history of diabetes to type 2 diabetes (DM), the independent influence of HIV on DM is still up for debate [11]. Circumstances that raise the likelihood of developing diabetes include hepatitis C virus (HCV) infection [10], particular drug abuse (e.g., corticosteroids), opiate use, and low testosterone [11]. ART-associated lipoatrophy [10] and buildup of visceral fat [9, 12] are other threatening agents for DM in HIV-infected persons, as are HIV-concomitant inflammation [13-14]. From this in-depth article we can learn more about the root causes of DM in HIV-positive people [15].

Pathophysiological hypotheses

**Figure 1:**
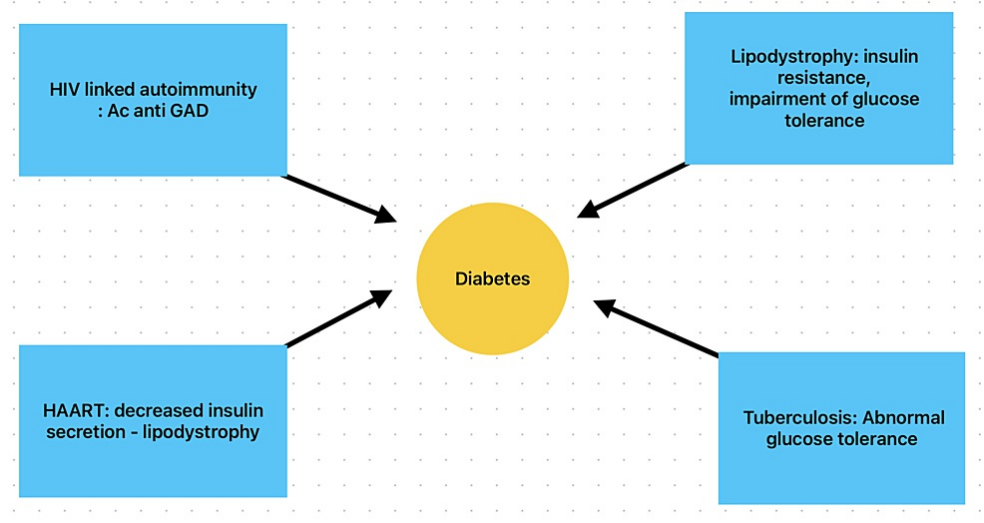
Pathophysiology of diabetes in HIV. HIV, human immunodeficiency virus; HAART, highly active antiretroviral therapy; anti-GAD, antibodies to glutamic acid decarboxylase Image credits: Authors

Numerous factors are theorized to contribute, including antiretroviral drugs, co-infection with HCV, lipodystrophy, mitochondrial dysfunction, elevated volume of circulating free fatty acids, increased accumulation of fats in muscles and organs, heightened levels of pro-inflammatory compounds, and a genetic predisposition (Figure 1). The specific importance of each factor in the process remains to be discovered.

Antiretroviral Therapy

Elevated blood sugar levels in HIV patients are typically caused by medical intervention. The effectiveness of HIV medication treatments can lead to two outcomes: either patients gain weight and experience improved well-being, or the drugs affect the body's glucose metabolism. Highly active antiretroviral therapy (HAART) has significantly improved clinical outcomes for HIV patients over the past two decades [16-17]. But it has been linked to metabolic problems like insulin resistance, cholesterol, and lipodystrophy. Protease inhibitors (PIs), like atazanavir, darunavir, saquinavir, and ritonavir, are the most crucial part of HAART. By getting in the way of glucose uptake receptors, especially GLUT-4 (glucose transporter type 4), these medicines have been shown to cause insulin resistance and lower insulin output. This causes insulin resistance, adipose tissue inflammation, and free fatty acids (FFAs) production. It is important to remember that not all PIs have the same effects on metabolism and that hyperglycemia caused by PIs usually goes away on its own in almost all patients when the drugs are stopped [18].

For example, lopinavir and ritonavir raise fasting levels of triglycerides and FFAs, but indinavir increases insulin resistance with minimal influence on lipid metabolism. Changes in first-phase insulin release and a 25% reduction in cell function were observed in HIV-infected people treated with nelfinavir, indinavir, lopinavir, and saquinavir for 12 weeks [19-20]. These results highlight the importance of evaluating the metabolic effects of PIs individually, and they show that PIs do not have a uniform effect on diabetes [20].

Nucleoside analogs are essential to HAART. Adjusted relative risk (RR) per year of NRTI (nucleoside reverse transcriptase inhibitor) exposure for stavudine was 1.20, p = 0.0001 in a meta-analysis of studies involving 130,151 person-years of exposure [21]. Stavudine was the most dangerous, although zidovudine and didanosine were also not without danger. Insulin resistance, lipodystrophy, and mitochondrial dysfunction have all been linked to long-term HIV therapy in humans [20].

Like other NNRTIs (non-nucleoside reverse transcriptase inhibitors), Efavirenz has been linked to a slight but observable rise in blood sugar levels compared to atazanavir [22].

*Lipodystrophy* 

Both HIV infection and therapy may cause metabolic problems, such as a shift in the location of fat cells. Modifications in the distribution of fat, hypertension, dyslipidemia, and abnormalities in glucose metabolism are all included in a consensual description of HIV lipodystrophy from the early 1995s [23]. Increased levels of inflammatory cytokines such as tumor necrosis factor (TNF) are linked to HIV lipodystrophy [24], and these cytokines have a role in insulin resistance, poor glucose tolerance, and perhaps the development of diabetes. Increased C-reactive protein and leptin levels and reduced adiponectin levels are common inflammatory and adipokine abnormalities in HIV-infected persons with metabolic syndrome [25] that might contribute to the development of diabetes.

Sixty-four percent of 116 patients treated with protease inhibitors for approximately 14 months were found to have lipodystrophy [26]. In contrast, only three percent of 32 patients who had never had protease inhibitor treatment showed signs of lipodystrophy. Lipodystrophy has also been linked to several transcriptase inhibitors, namely stavudine and zidovudine [27].

HIV/AIDS patients and diabetes management

*Metformin* 

Biguanides, including metformin, are a class of medicaments commonly used to cure diabetes, particularly type 2 diabetes. Metformin, in particular, is considered the preferred medicament for type 2 diabetes and has an undisputed safety record and effectiveness, as indicated in treatment guidelines. Metformin can lower HbA1c (glycated hemoglobin) levels by an average of 1% when used alone. It has several advantages, such as minimal risk of hypoglycemia when used singly, minimal weight gain, and reasonable cost. Additionally, metformin offers an added benefit for preventing cardiovascular disease (CVD) [28]. Alimentary tract symptoms such as nausea and diarrhea may occur as side effects of metformin due to its sensitivity to the gastrointestinal system. When metformin is taken with dolutegravir, a drug interaction can increase metformin concentration, potentially leading to a severe adverse effect called lactic acidosis. Therefore, caution should be exercised when taking both drugs simultaneously [29].

*Sulfonylureas* 

Increased insulin secretion from the pancreas is the key to the success of sulfonylureas like glipizide, glimepiride, glyburide, and micronized glyburide. Historically, sulfonylureas have been used safely and effectively, reducing HbA1c by 1%. The risks include hypoglycemia, failure, and weight gain (2-4 kg). There have been no previous studies on the relationship with HAART; nonetheless, HAART's apparent overall improvement may worsen the weight gain connected to sulfonylureas [30].

*Thiazolidinediones* 

Rosiglitazone and pioglitazone are two types of thiazolidinediones. They make it easier for target cells to respond to insulin, lowering glucose levels. Thiazolidinediones can bring down HbA1c by 1%. Pioglitazone lowers the chance of CVD by making high density lipoprotein (HDL) levels higher and cholesterol and liver fat levels lower [31-32]. The high price of thiazolidinediones and the possibility that they could make you more likely to get bladder cancer are both terrible things about them [33-34].

Insulin

Insulin is typically administered through injections and is the preferred medication for treating HIV-related diabetes. Unlike antiviral therapy, insulin does not interfere with the effectiveness of HIV treatment. In addition to its glucose-lowering effects, insulin has been found to lessen inflammation markers such as TNF-alpha. However, it is worth noting that newer insulin treatments, such as insulin analogs, can be expensive. It is essential to be aware that all forms of insulin can potentially lead to weight gain [30].

*Incretin Mimetics* 

Injectable therapies such as glucagon-like peptide 1 (GLP-1) receptor agonists are also available to treat diabetes. By increasing glucose-dependent insulin secretion, GLP-1 reduces insulin requirements and, by extension, postprandial glucagon secretion [35]. The use of GLP-1 analogs has several benefits, including a decrease in HbA1c by 1%, the absence of hypoglycemia, the promotion of weight loss, and the preservation of beta cell function. Their major drawbacks are high prices and uncomfortable abdominal side effects [30].

Glucagon-like peptide 1 and the dipeptidyl peptidase 4 (DPP-4) inhibitors sitagliptin and vidagliptin have a similar route; however, the DPP-4 inhibitors have fewer gastrointestinal (GI) side effects and do not significantly increase weight reduction. Having molecular targets on immune cells makes predicting these medications challenging for those living with HIV. A modest study evaluating this focused activity in sitagliptin-treated HIV-infected individuals found no changes in CD4 or HIV RNA counts [36]. When taking saxagliptin with cytochrome P450 3A4/5 inhibitors like ritonavir, the dosage needs to be decreased [37].

*Gliflozins and Meglitinides* 

Glucose reabsorption is inhibited and is excreted in the urine when the body processes SGLT2 (sodium-glucose cotransporter-2) inhibitors like dapagliflozin and canagliflozin. Weight loss, reduced blood pressure, and no hypoglycemia are only some benefits of gliflozins. Consequences of glycosuria, such as urinary tract infections and vaginal yeast infections, are among the drawbacks [38]. It is anticipated that dapagliflozin will have no interaction with ART. However, a higher dosage is required when canagliflozin is combined with ritonavir (canagliflozin dosing guidelines [39]).

Short-acting insulin-release stimulants are used to treat diabetes. They are costly, must be dosed often, and have a limited impact on HbA1c. As a result, they are employed in clinical settings less frequently [30].

**Table 1:** Various warning and precautions for anti-diabetic drugs in HIV. DPP-4, dipeptidyl peptidase 4; HIV, human immunodeficiency virus [37]

Drugs	Warnings and precautions
Metformin	Co-administration with dolutegravir could increase metformin concentration
Insulin	Weight gain
Sulfonylureas	Weight gain
DPP-4 inhibitors	Should be reduced when used in combination with cytochrome P450 3A4/5 inhibitors such as ritonavir
Gliflozins	Canagliflozin must be increased when co-administered with ritonavir

Glucose objectives

Patients with diabetes should strive for an HbA1c of 7%, which is equivalent to a mean blood glucose level of 152 mg/dL (8.4 mmol/L), fasting and preprandial readings of 129 mg/dL (7.4 mmol/L), and postprandial level of 185 mg/dL (10.2 mmol/L) [40]. Since HbA1c tends to underestimate hyperglycemia in HIV patients, healthcare providers caring for persons infected with the virus should use a more stringent target value when tracking the effectiveness of diabetes management. The macrovascular consequences of DM (coronary artery disease, cerebrovascular disease, and peripheral vascular disease) may be mitigated by attaining glycemic objectives, particularly in intensive remedy in newly diagnosed diabetics [41-42].

It has been established that vascular complications of DM (retinopathy, neuropathy, and nephropathy) are lessened when glycemic objectives are met [41, 43-44]. However, more significant severe hypoglycemia and mortality have been found in clinical studies due to more stringent glucose management [45]. This shows how important it is to modify one's objectives. More strict aims for DM therapy have been demonstrated to benefit individuals with low HbA1c at baseline (mild DM). Furthermore, stringent control (HbA1c 5.9%-6.6%) is more beneficial for younger, healthier persons, whereas looser control (HbA1c 7.4%-8.1%) may be more beneficial for senior patients with many comorbidities and a tendency to hypoglycemia [40].

Antiretroviral therapy for diabetic patients

It is best to leave the start of ART to experts in HIV management [45] due to the complexity of the treatment regimens, the well-established connection of certain drugs with the emergence of ailments of glucose metabolism, and the potential of certain drugs to result in significant medication interactions.

Nucleoside Reverse Transcriptase Inhibitors and Protease Inhibitors

The research found that in persons getting HIV treatment in a province of Canada in 2017 [17], the threat of acquiring type 2 diabetes in people living with HIV rises with prolonged use of stavudine (d4T) or first-generation protease inhibitors like nelfinavir or indinavir. Any time a patient's blood sugar level was 10.9 mmol/L or higher, their HbA1C level was 7% or above, they were provided anti-diabetic medicine, or they otherwise met the criteria for a diagnosis of diabetes, they were classified as having acquired type 2 diabetes. The chances of developing diabetes did not increase with either the age at which HIV was diagnosed or the age at which treatment began [17]. Diabetes was not associated with either gender, weight, or BMI at the time of diagnosis. Many HIV-related variables were linked with the development of diabetes, including a reduced CD4 T-cell nadir and a decreased CD4 cell count at the beginning of ART, for which data on viral load was available for a subset of the population (N = 1065) [18]. Patients who began medicaments with a non-nucleoside reverse transcriptase inhibitor (NNRTI)-based management had a lower risk of acquiring diabetes (p = 0.003) [18]. Patients who began treatment before 2005 had almost 50 times the chance of acquiring diabetes than those who started treatment between 2005 and 2009 [19]. Users of stavudine (d4T), zidovudine (AZT), lopinavir (Norvir), indinavir (Norvir), and nelfinavir (Norvir) over a longer length of time had a higher risk of developing diabetes, according to a univariate study. For every 10 days on nelfinavir, the chance of developing diabetes increased by 63%; for every 10 days on stavudine, the risk increased by 39% [22]. When patients were receiving antiretroviral medication, stavudine was given 21% of the time but only 7% of the time when they were not (p = 0.01) [19].

## Conclusions

Therefore, this study emphasizes the links between people with diabetes and HIV infection in many communities across the globe. Patterns in diabetes incidence over time show that HIV and ART are substantial contributors, yet these patterns fluctuate from generation to generation, demonstrating the uneven impact of ART. The epidemiological and pathophysiological picture is further complicated by the presence of co-infections, which are commonly seen in HIV-positive individuals. Anti-diabetic medicines should be carefully selected and monitored for potential comorbidities and drug interactions. PI-based regimens should not be used on people who are at an absurd probability of acquiring the disease.

## References

[1] DeFronzo RA, Ferrannini E, Alberti KGMM (2015). International Textbook of Diabetes Mellitus.

[2] International Diabetes Federation (2017). IDF diabetes atlas. Brussels, Belgium: International Diabetes Federation.

[3] (2023). Joint United Nations Programme on HIV/AIDS (UNAIDS). https://data.unaids.org/governance/pcb02/pcb_02_95_07_report_en.pdf.

[4] Atun R, Davies JI, Gale EAM (2017). Diabetes in sub-Saharan Africa: from clinical care to health policy. Lancet Diabetes Endocrinol.

[5] Pepin ME, Padgett LE, McDowell RE (2018). Antiretroviral therapy potentiates high-fat diet induced obesity and glucose intolerance. Mol Metab.

[6] White DL, Ratziu V, El-Serag HB (2008). Hepatitis C infection and risk of diabetes: a systematic review and meta-analysis. J Hepatol.

[7] Dooley KE, Chaisson RE (2009). Tuberculosis and diabetes mellitus: convergence of two epidemics. Lancet Infect Dis.

[8] Brown TT, Cole SR, Li X (2005). Antiretroviral therapy and the prevalence and incidence of diabetes mellitus in the multicenter aids cohort study. Arch Intern Med.

[9] De Wit S, Sabin CA, Weber R (2008). Incidence and risk factors for new-onset diabetes in hiv-infected patients. Diabetes Care.

[10] Rasmussen LD, Mathiesen ER, Kronborg G (2012). Risk of diabetes mellitus in persons with and without HIV: a Danish nationwide population-based cohort study. PLoS One.

[11] Polsky S, Floris-Moore M, Schoenbaum EE (2011). Incident hyperglycaemia among older adults with or at-risk for HIV infection. Antivir Ther.

[12] Triant VA, Lee H, Hadigan C (2007). Increased acute myocardial infarction rates and cardiovascular risk factors among patients with human immunodeficiency virus disease. J Clin Endocrinol Metab.

[13] Galli L, Salpietro S, Pellicciotta G (2012). Risk of type 2 diabetes among HIV-infected and healthy subjects in Italy. Eur J Epidemiol.

[14] Howard AA, Hoover DR, Anastos K (2010). The effects of opiate use and hepatitis C virus infection on risk of diabetes mellitus in the Women's Interagency HIV Study. J Acquir Immune Defic Syndr.

[15] Butt AA, McGinnis K, Rodriguez-Barradas MC (2009). HIV infection and the risk of diabetes mellitus. AIDS.

[16] Palella FJ Jr, Delaney KM, Moorman AC (1998). Declining morbidity and mortality among patients with advanced human immunodeficiency virus infection. HIV Outpatient Study Investigators. N Engl J Med.

[17] Larsson A, Larsson SE (1978). The effects of ethylene-1-hydroxy-1, 1-diphosphonate on cellular transformation and organic matrix of the epiphyseal growth plate of the rat--a light microscopic and ultrastructural study. Acta Pathol Microbiol Scand A.

[18] Samaras K (2012). The burden of diabetes and hyperlipidemia in treated HIV infection and approaches for cardiometabolic care. Curr HIV/AIDS Rep.

[19] Woerle HJ, Mariuz PR, Meyer C (2003). Mechanisms for the deterioration in glucose tolerance associated with HIV protease inhibitor regimens. Diabetes.

[20] Kalra S, Kalra B, Agrawal N (2011). Understanding diabetes in patients with HIV/AIDS. Diabetol Metab Syndr.

[21] De Wit S, Sabin CA, Weber R (2008). Incidence and risk factors for new-onset diabetes in HIV-infected patients: the Data Collection on Adverse Events of Anti-HIV Drugs (D:A:D) study. Diabetes Care.

[22] Erlandson KM, Kitch D, Tierney C (2014). Impact of randomized antiretroviral therapy initiation on glucose metabolism. AIDS.

[23] Carr A, Samaras K, Burton S (1998). A syndrome of peripheral lipodystrophy, hyperlipidaemia and insulin resistance in patients receiving HIV protease inhibitors. AIDS Lond Engl.

[24] Vigouroux C, Maachi M, Nguyên T-H (2003). Serum adipocytokines are related to lipodystrophy and metabolic disorders in hiv-infected men under antiretroviral therapy. AIDS Lond Engl.

[25] Samaras K (2009). Prevalence and pathogenesis of diabetes mellitus in HIV-1 infection treated with combined antiretroviral therapy. J Acquir Immune Defic Syndr.

[26] Carr A (2000). HIV protease inhibitor-related lipodystrophy syndrome. Clin Infect Dis.

[27] Zannou DM, Denoeud L, Lacombe K (2009). Incidence of lipodystrophy and metabolic disorders in patients starting non-nucleoside reverse transcriptase inhibitors in Benin. Antivir Ther.

[28] Lamanna C, Monami M, Marchionni N (2011). Effect of metformin on cardiovascular events and mortality: a meta-analysis of randomized clinical trials. Diabetes Obes Metab.

[29] (2023). Tivicay (dolutegravir) tablets for oral use. https://www.accessdata.fda.gov/drugsatfda_docs/label/2013/204790lbl.pdf.

[30] Monroe AK, Glesby MJ, Brown TT (2015). Diagnosing and managing diabetes in HIV-infected patients: current concepts. Clin Infect Dis.

[31] Mannucci E, Monami M, Lamanna C (2008). Pioglitazone and cardiovascular risk. A comprehensive meta-analysis of randomized clinical trials. Diabetes Obes Metab.

[32] Deeg MA, Buse JB, Goldberg RB (2007). Pioglitazone and rosiglitazone have different effects on serum lipoprotein particle concentrations and sizes in patients with type 2 diabetes and dyslipidemia. Diabetes Care.

[33] Lewis JD, Ferrara A, Peng T (2011). Risk of bladder cancer among diabetic patients treated with pioglitazone: interim report of a longitudinal cohort study. Diabetes Care.

[34] Schernthaner G, Currie CJ, Schernthaner GH (2013). Do we still need pioglitazone for the treatment of type 2 diabetes? A risk-benefit critique in 2013. Diabetes Care.

[35] Drucker DJ, Nauck MA (2006). The incretin system: glucagon-like peptide-1 receptor agonists and dipeptidyl peptidase-4 inhibitors in type 2 diabetes. Lancet Lond Engl.

[36] Goodwin SR, Reeds DN, Royal M (2013). Dipeptidyl peptidase IV inhibition does not adversely affect immune or virological status in HIV infected men and women: a pilot safety study. J Clin Endocrinol Metab.

[37] (2023). Onglyza® (saxagliptin) | adult type 2 diabetes medication. https://www.onglyza.com/type-2-diabetes-medication.html.

[38] Neal B, Perkovic V, de Zeeuw D (2013). Rationale, design, and baseline characteristics of the Canagliflozin Cardiovascular Assessment Study (CANVAS)--a randomized placebo-controlled trial. Am Heart J.

[39] (2023). Invokana®: the first sglt2i approved for initiation in adults with t2d and an egfr as low as 30 who have dkd,* established cvd, or are in need of glycemic control1-4. https://www.invokanahcp.com/dosing.

[40] Inzucchi SE, Bergenstal RM, Buse JB (2012). Management of hyperglycaemia in type 2 diabetes: a patient-centered approach. Position statement of the American Diabetes Association (ADA) and the European Association for the Study of Diabetes (EASD). Diabetologia.

[41] Holman RR, Paul SK, Bethel MA (2008). 10-year follow-up of intensive glucose control in type 2 diabetes. N Engl J Med.

[42] Abraira C, Colwell J, Nuttall F (1997). Cardiovascular events and correlates in the veterans affairs diabetes feasibility trial. veterans affairs cooperative study on glycemic control and complications in type II diabetes. Arch Intern Med.

[43] Gerstein HC, Miller ME, Byington RP (2008). Effects of intensive glucose lowering in type 2 diabetes. N Engl J Med.

[44] Patel A, MacMahon S, Chalmers J (2008). Intensive blood glucose control and vascular outcomes in patients with type 2 diabetes. N Engl J Med.

[45] Kelly TN, Bazzano LA, Fonseca VA (2009). Systematic review: glucose control and cardiovascular disease in type 2 diabetes. Ann Intern Med.

